# A modified Rapid Emergency Medicine Score incorporating C-reactive protein for mortality prediction in septic shock

**DOI:** 10.1590/1806-9282.20260890

**Published:** 2026-10-23

**Authors:** Serdar Özdemir, İbrahim Altunok, Abuzer Özkan, Hatice Şeyma Akça, Serkan Emre Eroğlu

**Affiliations:** 1 Ümraniye Training and Research Hospital, Department of Emergency Medicine – Istanbul, Turkey.; 2 Taksim Training and Research Hospital, Department of Emergency Medicine – Istanbul, Turkey.; 3 Karamanoğlu Mehmet Bey University, Department of Emergency Medicine – Karaman, Turkey.

**Keywords:** Septic shock, Mortality, Risk assessment, C-reactive protein, Emergency service

## Abstract

**OBJECTIVE:**

The aim of this study was to evaluate the prognostic performance of the Rapid Emergency Medicine Score incorporating C-reactive protein for predicting 30-day mortality in patients with septic shock and to compare its performance with the Rapid Emergency Medicine Score, National Early Warning Score, Modified Early Warning Score, quick Sequential Organ Failure Assessment, and Sequential Organ Failure Assessment.

**METHODS:**

This retrospective observational study included 124 adult patients diagnosed with septic shock in a tertiary emergency department. Rapid Emergency Medicine Score incorporating C-reactive protein was calculated as Rapid Emergency Medicine Score +(C-reactive protein /50), using an a priori clinically determined C-reactive protein scaling factor.

**RESULTS:**

The median age was 63 years, 52.4% of patients were female, and 76 patients (61.3%) died within 30 days. Rapid Emergency Medicine Score incorporating C-reactive protein had the highest numerical AUC (0.770), corresponding to moderate discrimination, followed by National Early Warning Score (0.755), Modified Early Warning Score (0.746), quick Sequential Organ Failure Assessment (0.688), Rapid Emergency Medicine Score (0.638), and Sequential Organ Failure Assessment (0.593). Rapid Emergency Medicine Score incorporating C-reactive protein had significantly higher AUCs than Rapid Emergency Medicine Score (ΔAUC=0.132, p<0.001) and Sequential Organ Failure Assessment (ΔAUC=0.177, p=0.049), whereas differences compared with National Early Warning Score, Modified Early Warning Score, and quick Sequential Organ Failure Assessment were not statistically significant. In the Firth-penalized logistic regression model, Rapid Emergency Medicine Score incorporating C-reactive protein remained associated with 30-day mortality (OR per 1-point increase, 1.22; 95%CI 1.11–1.36; p<0.001).

**CONCLUSION:**

Rapid Emergency Medicine Score incorporating C-reactive protein showed moderate discrimination for 30-day mortality in this exploratory development cohort but did not demonstrate superior discrimination compared with National Early Warning Score, Modified Early Warning Score, or quick Sequential Organ Failure Assessment. Because Rapid Emergency Medicine Score incorporating C-reactive protein was developed and evaluated in the same cohort, these findings should be considered hypothesis-generating. Independent external validation is required before its clinical utility can be established.

## INTRODUCTION

Sepsis and septic shock are among the clinical conditions frequently encountered in emergency departments, characterized by systemic inflammatory response and multiple organ dysfunction and associated with high mortality rates. Accurate risk stratification at an early stage is critically important for preventing a fatal course^1,2,3^. Therefore, there is a growing need for reliable, rapid, and applicable scoring systems to predict prognosis in the management of patients with sepsis^4,5^.

Various early warning systems developed for this purpose assist clinicians in patient monitoring and management. The National Early Warning Score (NEWS) allows dynamic monitoring of a patient’s clinical condition based on vital signs. While effective in predicting in-hospital adverse outcomes, its specificity may be limited for rapidly progressing conditions like sepsis^6,7,8^. Similarly, the quick Sequential Organ Failure Assessment (SOFA) score has been recommended as a rapid screening tool for diagnosing sepsis; however, several studies have reported its inadequate sensitivity and insufficiency in supporting early intervention decisions^9,10^.

Another early warning system is the Rapid Emergency Medicine Score (REMS), which includes parameters such as age, Glasgow Coma Scale (GCS) score, mean arterial pressure (MAP), heart rate, respiratory rate, and oxygen saturation^11^. REMS is widely used, particularly in emergency department settings^11^, due to its practicality and predictive power in certain clinical situations^12,13^. However, because REMS is based exclusively on physiological parameters and does not incorporate biomarkers reflecting the inflammatory response, its prognostic performance may be limited in conditions characterized by profound systemic inflammation, such as septic shock^14,15^. We speculated that integrating biomarkers that directly represent the inflammatory response into these scoring systems could result in more precise models. Based on this premise, REMS-C, a new score created by integrating the C-reactive protein (CRP) level into REMS, constituted the focal point of this study. REMS-C was hypothesized to provide a more comprehensive assessment by considering both the patient’s physiological stability and the level of inflammatory response.

This study aimed to determine the prognostic value of REMS-C in predicting 30-day mortality in patients presenting to the emergency department with septic shock and to evaluate its prognostic performance and compare it with REMS, NEWS, MEWS, and SOFA for predicting 30-day mortality.

## METHODS

### Study design and population

This retrospective observational study included adult patients diagnosed with septic shock and managed in the emergency department of a tertiary university hospital between June 1, 2021, and June 1, 2022.

### Inclusion and exclusion criteria

Patients aged 18 years or older who met the Sepsis-3 criteria for septic shock at emergency department presentation were eligible for inclusion. Septic shock was defined as persistent hypotension requiring vasopressor support to maintain a MAP ≥65 mmHg and a serum lactate level >2 mmol/L despite adequate fluid resuscitation. Patients with incomplete clinical, laboratory, or outcome data were excluded from the study.

### Data collection and score calculation

Demographic characteristics (age and sex), vital signs (heart rate, respiratory rate, systolic blood pressure, MAP, oxygen saturation, and body temperature), neurological status (GCS score and AVPU scale), laboratory parameters, and outcome data were retrospectively obtained from the hospital information management system and electronic medical records. Laboratory variables included pH, lactate, complete blood count parameters, inflammatory biomarkers, and routine biochemical measurements recorded at the time of emergency department admission.

REMS-C was developed as an exploratory modification of REMS by incorporating CRP as a marker of inflammatory burden. The CRP scaling factor was determined a priori on clinical grounds, before outcome analyses were performed. The upper reference limit for CRP at our institution is approximately 5 mg/L; therefore, 50 mg/L, corresponding to approximately 10 times this reference limit, was selected as a pragmatic and clinically interpretable increment. Accordingly, each 50-mg/L increase in CRP contributed one additional point to the modified score. This scaling factor was not derived from regression coefficients or selected by testing alternative weights to optimize predictive performance in the study cohort. REMS-C was calculated as follows: REMS-C=REMS+(CRP/50).

### Statistical analysis

Statistical analyses were performed using Jamovi software (version 2.6.26.0; The Jamovi Project, Sydney, Australia). The distribution of continuous variables was assessed using the Shapiro-Wilk test. Continuous variables were presented as median and interquartile range (IQR, 25th–75th percentile), whereas categorical variables were presented as frequencies and percentages. Comparisons between survivor and non-survivor groups were performed using the Mann-Whitney U test for continuous variables and the chi-square test or Fisher’s exact test, as appropriate, for categorical variables.

In an additional exploratory analysis, restricted cubic spline modeling with three knots at the 10th, 50th, and 90th percentiles of the CRP distribution was used to evaluate potential nonlinearity in the association between CRP and 30-day mortality. The spline model was compared with the corresponding linear model using a likelihood-ratio test.

Prognostic discrimination was evaluated using receiver operating characteristic (ROC) curve analysis, and differences between AUC values were assessed using the DeLong test.

To examine whether REMS-C was associated with 30-day mortality after adjustment for selected clinically relevant covariates while reducing small-sample bias, Firth-penalized logistic regression was performed. The model was specified parsimoniously and included REMS-C as the predictor of primary interest together with sex and serum lactate, which were selected on clinical grounds and are not components of REMS-C. Individual physiological variables incorporated within REMS-C were not entered simultaneously into the model to avoid predictor redundancy. Odds ratios (ORs) with 95%CIs were reported.

All statistical tests were two-tailed, and a p-value <0.05 was considered statistically significant.

## RESULTS

A total of 124 patients diagnosed with septic shock were included in the study. Of these patients, 52.4% (n=65) were female, and the median age was 63 years (25th–75th percentile: 47–77). The 30-day mortality rate was 61.3% (n=76).

Univariate analyses revealed statistically significant differences between survivors and non-survivors across several clinical and laboratory parameters. Female sex, lower systolic and MAP, higher heart rate, higher respiratory rate, lower oxygen saturation, lower lymphocyte count, higher CRP levels, and higher REMS, NEWS, MEWS, and REMS-C scores were significantly associated with mortality. Detailed comparisons between the survivor and mortality groups are presented in Table 1.

**Table 1:** Descriptive characteristics of patients with septic shock and comparison between survivor and mortality groups.

Parameters	Total n=124	Survivor group n=48 (39%)	Mortality group n=76 (61%)	p-value
Age (years)	63 (47–77)	64 (57–76)	62 (39–78)	0.22
Gender				
Male	59 (47.6)	32 (66.7)	27 (35.5)	**<0.001**
Female	65 (52.4)	16 (33.3)	49 (64.5)	
Systolic blood pressure (mmHg)	77 (66–85)	81 (74–88)	70 (62–80)	**<0.001**
Diastolic blood pressure (mmHg)	45 (38–50)	48 (40–53)	44 (37–50)	0.019
Heart rate (beats per minute)	116 (102–130)	106 (90–121)	120 (110–133)	**<0.001**
Body temperature (°C)	37.00 (36.70–37.70)	36.90 (36.68–37.73)	37.00 (36.70–37.62)	0.78
Respiratory rate (breaths per minute)	24 (18–28)	19 (16–25)	25 (21–29)	**<0.001**
Mean arterial pressure (mmHg)	55 (48–62)	60 (51–64)	52 (45–60)	**<0.001**
Oxygen saturation (%)	89 (84–95)	92 (89–96)	86 (82–95)	0.001
Glasgow Coma Scale score	15 (12–15)	15 (14–15)	14 (10–15)	**0.095**
Rapid Emergency Medicine Score	9 (6–11)	7 (5–10)	10 (7–12)	**0.010**
National Early Warning Score	9 (7–11)	7 (5–10)	10 (8–11)	**<0.001**
Modified Early Warning Score	6 (4–8)	5 (3–6)	7 (5–8)	**<0.001**
pH	7.36 (7.27–7.42)	7.39 (7.29–7.44)	7.31 (7.24–7.40)	**0.017**
C-reactive protein (mg/L)	212 (116–296)	111 (68–183)	266 (193–341)	**<0.001**
Amylase (U/L)	39 (24–80)	50 (30–93)	33 (22–55)	**0.033**
SOFA score	8.00 (6.75–10.00)	8.00 (6.00–9.00)	9.00 (7.00–10.00)	0.14
REMS-C	13.2 (9.3–16.8)	9.5 (7.8–12.5)	15.7 (12.2–17.8)	**<0.001**

SOFA: Sequential Organ Failure Assessment; pCO_2_: partial pressure of carbon dioxide; REMS-C: Rapid Emergency Medicine Score incorporating C-reactive protein. Bold values indicate statistically significant p-values (p<0.05).

Restricted cubic spline analysis showed no statistically significant evidence of nonlinearity in the association between CRP and 30-day mortality after adjustment for REMS (p for nonlinearity=0.164).

The prognostic performances of REMS-C, NEWS, MEWS, qSOFA, REMS, and SOFA for predicting 30-day mortality were evaluated using ROC curve analysis (Figure 1 and Table 2). REMS-C yielded an AUC of 0.770 (95%CI 0.681–0.858), while the AUCs were 0.755 for NEWS, 0.746 for MEWS, 0.688 (95%CI 0.597–0.779) for qSOFA, 0.638 for REMS, and 0.593 for SOFA. Pairwise DeLong analysis showed significantly higher AUCs for REMS-C than for REMS (ΔAUC=0.132, p<0.001) and SOFA (ΔAUC=0.177, p=0.049), whereas the differences compared with NEWS (p=0.754), MEWS (p=0.556), and qSOFA (ΔAUC=0.082, p=0.119) were not statistically significant.

**Figure 1:**
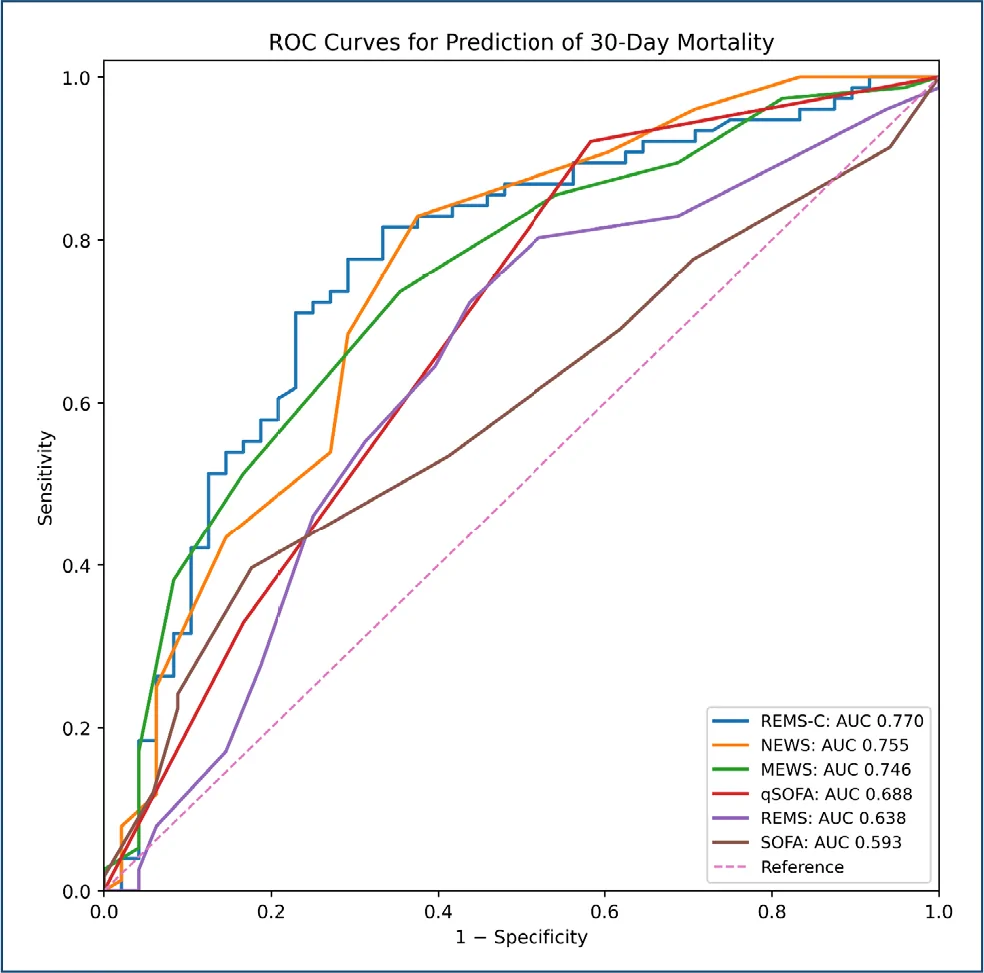
Receiver operating characteristic curves of Rapid Emergency Medicine Score incorporating C-reactive protein, National Early Warning Score, Modified Early Warning Score, quick Sequential Organ Failure Assessment, Rapid Emergency Medicine Score, and Sequential Organ Failure Assessment for predicting 30-day mortality in patients with septic shock.

**Table 2:** Prognostic performance of scoring systems for 30-day mortality.

Scoring system	AUC (95%CI)	Optimal cut-off	Sensitivity, %	Specificity, %	PPV, %	NPV, %
REMS-C	0.770 (0.681–0.858)	≥11.74	77.63	70.83	80.82	66.67
NEWS	0.755 (0.664–0.846)	≥8	82.89	62.50	77.78	69.77
MEWS	0.746 (0.657–0.834)	≥6	73.68	64.58	76.71	60.78
qSOFA	0.688 (0.597–0.779)	≥2	92.11	41.67	71.43	76.92
REMS	0.638 (0.534–0.742)	≥8	72.37	56.25	72.37	56.25
SOFA	0.593 (0.476–0.709)	≥10	39.66	82.35	79.31	44.44

AUC: area under the receiver operating characteristic curve; CI: confidence interval; MEWS: Modified Early Warning Score; NEWS: National Early Warning Score; NPV: negative predictive value; PPV: positive predictive value; qSOFA: quick Sequential Organ Failure Assessment; REMS: Rapid Emergency Medicine Score; REMS-C: Rapid Emergency Medicine Score–C-reactive protein; SOFA: Sequential Organ Failure Assessment.

Pairwise comparisons of AUC values using the DeLong method demonstrated that REMS-C significantly outperformed both REMS (ΔAUC=0.132, p<0.001) and SOFA (ΔAUC=0.177, p=0.049). However, no statistically significant differences were observed between REMS-C and NEWS (p=0.754) or between REMS-C and MEWS (p=0.556). Likewise, NEWS, MEWS, and SOFA did not significantly differ from one another in pairwise comparisons.

In the Firth-penalized logistic regression model including REMS-C, sex, and serum lactate, REMS-C remained associated with 30-day mortality (OR per 1-point increase, 1.22; 95%CI 1.11–1.36; p<0.001). Female sex was also associated with mortality (OR 3.00; 95%CI 1.30–6.91; p=0.010), whereas serum lactate was not independently associated with mortality (OR per 1 mmol/L, 1.04; 95%CI 0.89–1.21; p=0.604).

## DISCUSSION

In this study, we evaluated the prognostic performance of REMS-C, a modified version of REMS incorporating CRP, for predicting 30-day mortality in patients presenting to the emergency department with septic shock. REMS-C yielded the highest numerical AUC (0.770) among the evaluated scores, corresponding to moderate discrimination. Although its AUC was significantly higher than those of REMS and SOFA, no statistically significant differences were observed compared with NEWS, MEWS, or qSOFA. Therefore, the present findings do not establish superiority of REMS-C over these widely used early warning scores. Rather, they provide exploratory evidence that incorporation of CRP into REMS warrants further investigation.

The operating characteristics of REMS-C also warrant cautious interpretation. At the identified cut-off of ≥11.74, sensitivity was 77.63%, specificity was 70.83%, PPV was 80.82%, and NPV was 66.67%. These values are insufficient to support REMS-C as a stand-alone rule-in or rule-out tool for individual clinical decisions. In addition, unlike NEWS and MEWS, REMS-C requires an available CRP measurement. Although CRP is commonly obtained during the evaluation of patients with suspected sepsis, the present study did not assess laboratory turnaround time, cost-effectiveness, workflow impact, or whether REMS-C-guided risk classification improves clinical outcomes. Therefore, its potential incremental clinical utility over purely physiological scores remains to be established prospectively.

In recent years, various early warning scoring systems have been developed and comparatively evaluated for the diagnosis and prognosis of sepsis in emergency departments. REMS has garnered increasing attention for this purpose. In a retrospective study evaluating data from 1,622 patients, REMS was reported to predict in-hospital mortality with significantly greater accuracy, with an AUC value of 0.62, compared to sepsis-specific scores, such as quick SOFA and Systemic Inflammatory Response Syndrome, and demonstrated comparable performance to NEWS^16^. Similarly, in another study involving 225 patients with sepsis in the emergency department, REMS yielded an AUC value of 88.6%, significantly higher than that of the Simple Clinical Score^17^. These findings underscore REMS as a valuable tool, especially due to its ease of use by frontline clinicians such as nurses. Nevertheless, in a prospective study involving patients receiving early goal-directed therapy for sepsis, the predictive power of REMS was found to be inferior to the Mortality in Emergency Department Sepsis score (AUC: 0.62 vs. 0.74), although it outperformed the CURB-65 score^18^. Previous studies have demonstrated the utility of REMS in sepsis, with reported AUC values ranging from 0.62 to 0.89^19,20^. However, because REMS relies exclusively on physiological variables, it may not adequately reflect the inflammatory burden of septic shock. By incorporating CRP, REMS-C combines physiological and inflammatory information while maintaining bedside simplicity.

## LIMITATIONS

This study has several limitations. First, its retrospective design precludes causal inference and may introduce selection bias. Second, the study was conducted at a single center and included a relatively limited number of patients, which may restrict the generalizability of the findings. Most importantly, REMS-C was developed and evaluated in the same cohort without an independent validation sample. Consequently, the reported discrimination represents apparent performance and may overestimate performance in new populations. Furthermore, the CRP scaling factor was determined a priori on pragmatic clinical grounds rather than derived from statistical modeling; therefore, the selected weighting should not be interpreted as an optimized or validated prognostic weight. The present findings should consequently be regarded as exploratory and hypothesis-generating. In addition, although REMS-C had a significantly higher AUC than REMS and SOFA, its discrimination was not significantly different from that of NEWS, MEWS, or qSOFA; therefore, its incremental clinical utility over existing scoring systems remains uncertain. Independent external validation in larger, prospective, multicenter cohorts with different demographic characteristics, comorbidity profiles, and infection sources is required before the clinical utility of REMS-C can be established.

## CONCLUSION

In this exploratory proof-of-concept study, REMS-C showed moderate discrimination for 30-day mortality and had a higher numerical AUC than the other evaluated scores. Its discrimination was significantly higher than that of REMS and SOFA but not significantly different from NEWS, MEWS, or qSOFA. These findings do not establish incremental clinical utility or support REMS-C as a stand-alone bedside decision tool. Independent external validation and prospective assessment of clinical utility are required before its role in risk stratification can be determined.

## Data Availability

The datasets generated and/or analyzed during the current study are available from the corresponding author upon reasonable request.
